# An Uncommon Cause of Acute Encephalopathy in Multiple Myeloma

**DOI:** 10.7759/cureus.20605

**Published:** 2021-12-22

**Authors:** Jawaher Al Zeyoudi, Aisha Al Naqbi, Deanne Kashiwagi, Mustaqeem Siddiqui

**Affiliations:** 1 Internal Medicine, Sheikh Khalifa Medical City, Abu Dhabi, ARE; 2 Internal Medicine, Sheikh Shakbout Medical City, Abu Dhabi, ARE; 3 Hematology/Oncology, Sheikh Shakbout Medical City, Abu Dhabi, ARE

**Keywords:** altered mental status, hematologic malignancies, hyperammonemia, encephalopathy, multiple myeloma

## Abstract

Multiple myeloma commonly presents with bone pain, pathological fractures, hypercalcemia, anemia, and acute kidney injury. Altered mental status due to multiple myeloma is generally attributed to uremia, hypercalcemia, and hyperviscosity. In this report, we present a rare case of altered mental status due to high serum ammonia levels in a patient with advanced multiple myeloma and with no liver dysfunction.

## Introduction

Multiple myeloma is the second most common hematologic malignancy in the world [1]. Its incidence has been on the rise, with 34,920 new cases and 12,410 deaths estimated to occur in the US in 2021 [2]. It is found in the spectrum of plasma cell dyscrasias, which ranges from monoclonal gammopathy of unknown significance to plasma cell leukemia and extra-medullary multiple myeloma [3].

Typical manifestations of multiple myeloma include low blood counts (leukopenia, anemia, and thrombocytopenia), bone and calcium abnormalities (lytic bone lesions, pathological fractures, and hypercalcemia), infections (due to cytopenias and hypogammaglobulinemia), and kidney injury and failure [3]. Altered mental status is generally attributed to metabolic causes like uremia, kidney failure, hyperviscosity, and hypercalcemia. High ammonia levels in patients with multiple myeloma causing altered mental status in the absence of liver disease is a rare cause of encephalopathy and a diagnostic challenge [4].

## Case presentation

A 58-year-old woman with a medical history of type two diabetes mellitus, dyslipidemia, and multiple myeloma diagnosed four years ago presented to the emergency department complaining of dysuria and fever. She had completed nine cycles of VRd (bortezomib, lenalidomide, and dexamethasone) with denosumab. However, her disease had progressed further in one year and required additional six cycles of bendamustine and lenalidomide. Her last chemotherapy session with carfilzomib, dexamethasone, and daratumumab had been five months prior to her presentation. Her chemotherapy sessions were stopped following her admission to the hospital with severe acute respiratory syndrome coronavirus 2 (SARS-CoV-2) pneumonia.

In the ED, her physical examination was unremarkable. Laboratory results showed leukocyte counts of 8.12 x 10^9^/L, a hemoglobin level of 88 g/L, serum creatinine level of 140 micromol/L, serum urea of 4.60 mmol/L, and C-reactive protein of 69.26 mg/L. Urine analysis showed positive leukocyte esterase and no nitrates. CT abdomen and pelvis showed marked right perinephric fat stranding and patchy ill-defined parenchymal hypodensities raising the possibility of pyelonephritis. The patient was admitted to the general medical ward and was started on ceftriaxone.

**Figure 1:**
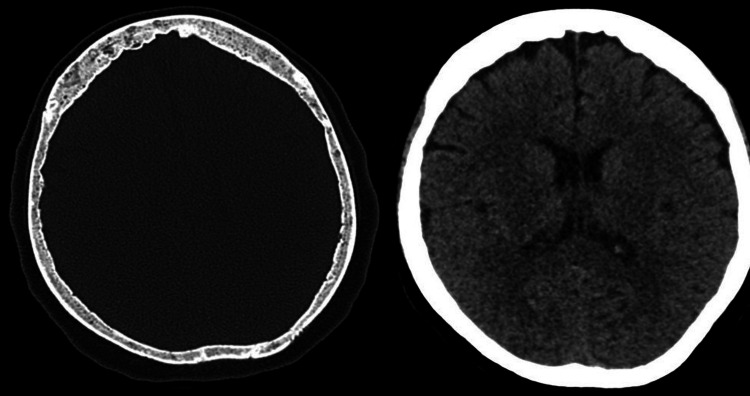
CT head without contrast showing normal gray-white matter differentiation of both cerebral hemispheres, and multiple small well-defined lytic lesions of the skull vault CT: computed tomography

Two days after the admission, the patient was noted to be confused, and her mental status started to deteriorate. Physical examination did not show any neurological deficit. CT scan of the head showed multiple small lytic lesions of the skull vault consistent with her advanced multiple myeloma disease (Figure 1).

Her extended electrolyte panel was unremarkable except for mild hypercalcemia (calcium corrected to albumin was 2.6 mmol/L). Her thyroid function tests were normal and her kidney function tests showed elevated creatinine of 160 micromol/L and normal urea. Arterial blood gas showed pH of 7.43, CO_2_ of 33.3 mmHg, O_2_ of 93.3 mmHg, and lactic acid of 0.5 mmol/L. A protein panel was ordered and showed high alpha globulins and kappa light chains (Table 1).

**Table 1:** Protein panel showing high alpha globulins and kappa light chains

Laboratory test	Value	Reference range	Units
Total protein	63	63–82	g/L
Alpha 1 globulins	2.6	1.0–2.3	g/L
Alpha 2 globulins	12	5.0–9.0	g/L
Beta globulins	4.3	4.3–6.8	g/L
Kappa	38	3.3–19.4	mg/dL
Lambda	2.13	5.7–26.3	mg/dL

The patient’s mental status continued to decline during the admission. Electroencephalography showed epileptiform discharges from the bilateral frontal central regions, and levetiracetam 1000 mg twice daily was then started as advised by the neurology team.

After one week of hospitalization, the patient’s Glasgow Coma Scale (GCS) score dropped to 9/15 and she became unresponsive. Her airway remained patent. Urgent MRI brain did not show any evidence of ischemic or hemorrhage stroke. The infectious disease team started the patient empirically on ampicillin and acyclovir, in addition to ceftriaxone to cover for bacterial meningitis. Lumbar puncture was not suggestive of an infective etiology. cerebrospinal fluid (CSF) culture showed no growth. The paraneoplastic syndrome panel was also sent from the CSF sample and came back negative. Antibiotics were reduced to ceftriaxone only to continue the course for pyelonephritis and urine culture grew non-extended spectrum beta-lactamase (ESBL) *Escherichia coli (E. coli)*.

Serum ammonia level was critically high with a value of 262 micromol/L. The patient had no prior history of liver disease. Her aspartate transaminase (AST) and alanine transaminase (ALT) were 26 IU/L and 10 IU/L respectively, and alkaline phosphatase (ALP) was 79 IU/L. She was started on oral and rectal lactulose with no significant improvement in mental status or reduction in serum ammonia levels. Ultrasound and CT abdomen did not show any signs of chronic liver disease or thrombosis. Rifaximin was added and hemodialysis was started as recommended by the nephrology team. However, despite undergoing five sessions of hemodialysis, her ammonia levels remained critically elevated and her GCS score did not improve.

The patient’s chemotherapy sessions were resumed once the liver disease was excluded, and a diagnosis of multiple myeloma-induced hyperammonemic encephalopathy was made after excluding other causes of encephalopathy. Her mental status improved significantly and she became fully alert three days after starting the chemotherapy. She continued to have three cycles of chemotherapy during her hospital stay. The ammonia level dropped to 99 micromol/L. The patient’s functional status continued to improve and she was discharged to a rehabilitation center for physical therapy.

## Discussion

High serum ammonia levels causing encephalopathy in patients with multiple myeloma and with no liver disease is very rare. The mechanism is poorly understood. However, in vitro studies have illustrated that myeloma cells in culture can produce excess ammonia due to amino acid metabolism [5]. The fact that hyperammonemia usually occurs in progressive forms of multiple myeloma also suggests a direct link between malignant plasma cells and ammonia levels. Other proposed mechanisms include the creation of a systemic-portal shunt due to plasma cells infiltration of the liver, the interference of plasma cells with urea metabolism, and excess ammonia production that accompanies the excess protein synthesis in myeloma cells [4].

Effective and early management of multiple myeloma can quickly decrease the serum ammonia level and normalize the mental status of patients. Upgrading the doses of the chemotherapy or using second-line agents might be required for resistant cases. Lora-Tamayo et al. have reported that 22 out of 25 patients in their study had lower serum ammonia levels when given combined and aggressive chemotherapy, with 15 patients out of the 22 ultimately surviving the encephalopathy. The overall mortality rate was 44% [6]. Pharm et al. have reported similar findings, with a 40% inpatient mortality in patients who were treated with chemotherapy and a 75% mortality in those who were not [7].

These studies show that hyperammonemic encephalopathy is a severe complication of multiple myeloma and is associated with high mortality. It should be considered in any patient with multiple myeloma and altered mental status. Chemotherapy seems to be the most helpful treatment in order to normalize ammonia level and clinical status.

## Conclusions

Our case illustrates hyperammonemic encephalopathy as a rare cause of altered mental status in patients with multiple myeloma. Early recognition of this cause and early management with chemotherapy is important since prognosis is poor and inpatient mortality is high. Physicians need to keep the diagnosis in mind with every multiple myeloma patient presenting with altered mental status. Further studies are needed to identify patients at risk and guide management.
